# How to Detect Porcine Endogenous Retrovirus (PERV) Infections in Patients After Transplantation of Pig Organs

**DOI:** 10.1111/xen.70028

**Published:** 2025-02-24

**Authors:** Joachim Denner, Hina Jhelum, Jinzhao Ban, Ludwig Krabben, Benedikt B. Kaufer

**Affiliations:** ^1^ Institute of Virology Free University Berlin Berlin Germany

**Keywords:** immunological methods, microchimerism, PCR methods, porcine endogenous retroviruses, virus safety, Western blot analysis

## Abstract

Porcine endogenous retroviruses (PERVs) are integrated into the genome of all pigs and can infect human cells in culture. However, no PERV infections have been reported in recipients following preclinical or clinical xenotransplantation or deliberate infection experiments. Detection of PERV infection in transplanted recipients is challenging due to microchimerism, such as the presence of pig cells containing PERV proviruses in the recipient. Based on our previous publications on PERV detection in xenotransplant recipients, particularly from the first clinical trials, we developed a comprehensive strategy to screen for PERV infections. Recipients can be monitored for increasing levels of viral genomic RNA and mRNA using real‐time reverse transcriptase (RT)‐PCR, which can indicate PERV expression and replication. To test this strategy, explanted pig hearts and organs from baboons after pig heart transplantation were analyzed. No PERV genomic RNA or mRNA was detected in these tissues, although both were found in PERV‐producing human control cells. Screening for antibodies against PERV as indirect evidence of infection is the method of choice. Recombinant viral proteins were prepared for use in Western blot assays. Animal antisera generated through immunization with recombinant PERV proteins served as positive controls. No antibodies against PERV were detected in transplanted baboons, even though microchimerism was observed in many of the animals' organs. For effective antibody screening, at least two PERV proteins should be used as antigens.

## 1 Introduction

Three different types of porcine endogenous retroviruses (PERVs) are found integrated in the genome of pigs: PERV‐A and PERV‐B are present in all pigs, PERV‐C is found in many, but not all pigs. PERV‐A and PERV‐B are able to infect human cells in cell cultures, whereas PERV‐C infects only pig cells [1]. In addition, PERV‐A can recombine with PERV‐C; the recombinant PERV‐A/C can infect human cells and is characterized by high replication rates [2, 3]. Since PERVs are integrated in the pig genome, they cannot be eliminated like other viruses from pig breeds. Attempts have been made to inactivate all proviruses in the pig genome using CRISPR/Cas [4, 5]. However, it remains uncertain whether this strategy is necessary, as no PERV transmission has been observed in any preclinical or clinical trials. Furthermore, off‐target effects of CRISPR/Cas are a potential concern, and breeding these animals into large colonies may present significant challenges. CRISPR/Cas‐treated pig cells were still capable of releasing intact viral particles [6], which are expected to contain viral genomic RNA with an inactivated reverse transcriptase (RT) sequence. It cannot be excluded that these particles might enter human cells, as they carry functional envelope proteins. Although the inactivated RT prevents the viral RNA from being transcribed into DNA, human cells express RT, either from LINE sequences [7] or human endogenous retroviruses (HERVs) [8]. Therefore, it cannot be ruled out that these RTs could potentially rescue PERV, facilitating reverse transcription and integration.

Retroviruses can induce tumors and immunodeficiencies in numerous species, including humans. Therefore, PERVs pose a unique risk for xenotransplantation using pig cells, tissues, or organs. The viruses most closely related to PERVs, for example, the murine leukemia viruses (MuLV), the feline leukemia viruses (FeLV), and the koala retrovirus (KoRV), induce leukemia, lymphoma, and immunodeficiencies in their natural host. However, there are no reports of these viruses causing diseases in other species [9, 10, 11].

No PERV infection was observed in non‐human primate (NHP) recipients of pig xenotransplants as well as in the first human patients receiving pig islet cells (for review, see Denner [12]). However, NHPs commonly used for preclinical trials of xenotransplantation do not represent an ideal animal model to assess the risk of PERV transmission. Rhesus macaques, cynomolgus macaques, and baboons were found to have a genetic deficiency in PERV‐A receptor 1 (PAR‐1), the main receptor for PERV entry [13]. This also explains why PERV was not transmitted in inoculation experiments in NHPs using high doses of PERV and triple immunosuppression [14]. PERV was also not transmitted when islet cells derived from Göttingen minipigs were transplanted into non‐diabetic cynomolgus monkeys using a macroencapsulation device, as shown by a Western blot analysis looking for PERV‐specific antibodies [15].

PERV was also not transmitted to the recipient when islet cells from Auckland Island pigs were transplanted into human patients for the treatment of diabetes. However, the pig islet cells were encapsulated and no immunosuppressive drugs were applied [16, 17].

When PBMCs of the Baltimore patient receiving the first pig heart were assayed for evidence of PERV integration, no indication of PERV was reported ([18], appendix p. 18). However, the results are unclear, especially because microchimerism was not detected in this assay.

In order to detect and characterize PERV in donor pigs, PCR‐based detection methods were developed. All integrated PERVs can be detected by performing conventional or real‐time PCR using primers binding to a highly conserved region of the polymerase sequence (PERVpol). If specific primers binding to the receptor‐binding sites in the envelope genes were used, PERV‐A, PERV‐B, PERV‐C, and PERV‐A/C can be discriminated. Conventional and real‐time RT‐PCRs detect expression of the provirus as mRNA or genomic viral RNA; immunofluorescence or immunohistochemistry detect expression at the protein level, RT‐assays and electron microscopy detect particle release, infection assays using human or pig cells detect infectivity, and determine the tropism of the virus (for review see Denner and Tönjes [1]).

The same methods can be applied for screening the recipient [19]. However, there are numerous difficulties when screening for PERV infection in recipients. The greatest difficulty is the presence of pig cells in most organs of the recipient, called microchimerism. Microchimerism is common in allotransplantation and pregnancy and was also observed in xenotransplantation [20]. PERV proviruses were found in all investigated organs of baboons after the transplantation of pig hearts [21, 22]. Highly sensitive PCR methods detecting short interspersed nuclear sequences (SINEs), which are present in hundreds of thousands of copies in the pig genome, allow us to demonstrate that the detected PERV proviruses are part of the genome of disseminated pig cells [22]. Other authors have developed a PCR assay to detect pig‐specific mitochondrial DNA (mtDNA), for example, mitochondrially encoded cytochrome c oxidase II (COX2) sequences as a marker of pig cells [23]. This assay, however, is less sensitive compared with the SINE assay. Therefore, different methods should be used to detect real PERV infections in recipients transplanted with pig cells or organs, which will be described here.

## 2 Methods

### 2.1 RNA Isolation, Real‐Time RT‐PCR

RNA was isolated from various tissues (liver, lung, kidney, spleen) of baboon recipients of pig hearts as well as of the left ventricle of an explanted pig heart using RNeasy plus mini kit (Qiagen, Hilden, Germany). Six baboons were investigated (A, B, C, D, E, and F). Liver, lung, kidney, and spleen samples were available from animal A, B, and C. From animal C the explanted pig heart was available (Table 1). The survival times were 197 days for animal A, 195 days for animal B and 169 days for animal C. The donor pigs were triple genetically modified: GGTA1‐KO (knockout of the porcine GGTA1 gene, which encodes for the 1,3‐galactosyltransferase that synthesizes the Gal epitopes), hCD46 (expression of the human CD46, also called membrane cofactor protein, MCP), and hTBM (expression of the human thrombomodulin) [24]. Animals A, D (14 days survival time), E (90 days survival time), and F (50 days survival time) were tested using a Western blot assay (see below). RNA was also isolated from 293 cells producing PERV‐A/C [25]. RNA concentration was determined using NanoDrop ND‐1000 (Thermo Fischer Scientific Inc., Worcester, MA, USA). In all baboon organs pig cells had been detected using real‐time PCR specific for porcine GAPDH and SINE sequences [22]. In order to control for absence of PERV DNA in the RNA preparation, a polymerase chain real‐time (PCR) was performed using specific primers and probes (Table 2). In order to analyze PERV‐pol expression, a real‐time RT‐PCR was performed using SensiFAST Probe No‐ROX One‐Step kit (Meridian Bioscience, Cincinnati, OH, USA). Briefly, cDNA was synthesized at 50°C for 30 min followed by initial denaturation at 95°C for 5 min and 45 amplification cycles of denaturation at 95°C for 15 s, annealing at 62°C for 30 s and extension at 72°C for 30 s.

### 2.2 Recombinant PERV Proteins

A sequence corresponding to the ectodomain of the transmembrane envelope protein p15E (amino acids 488–597) had been amplified by polymerase chain reaction from DNA from PK15 cells and cloned into the pCal‐n vector (Agilent, Santa Clara, CA, USA) [26]. The presence of the insert was verified by PCR and sequencing. *E. coli* BL21 DE3 cells were transformed with the construct. Recombinant p15E, which was N‐terminally fused to a 4 kDa calmodulin‐binding protein (CBP), was produced by induction with 0.2 mM isopropyl‐D‐thiogalactopyranoside (IPTG) when the *E. coli* culture reached an OD_600_ of 0.6. Cells were incubated for 3 h by shaking at 200 rpm and 37°C, harvested at 6000 × *g*, washed 2× with PBS, and frozen. Cells were lysed by incubation in 50 mM Tris/HCl pH 7.5, 150 mM NaCl, 2 mM CaCl_2,_ 10 mM DTT with 1 mg/mL lysozyme, 1000 U benzonase and complete protease inhibitor EDTA‐free (Roche, Mannheim, Germany) for 30 min at room temperature and then 20 s sonification cycles with 1 min brake on ice. The suspension was diluted with one volume binding buffer (50 mM Tris/HCL pH 7.5, 150 mM NaCl, 2 mM CaCl_2_) and centrifuged for 20 min at 50 000 × *g*. The protein was purified by calmodulin resin affinity chromatography using an ÄKTAprime plus unit (both Cytiva Life Sciences, Freiburg, Germany) according to the manufacturer protocol with 5 mM DTT in the binding and 2 mM DTT in the washing and elution (50 mM Tris/HCl pH 7.5, 150 mM NaCl, 2 mM EGTA) buffers. Recombinant gp70, comprising the entire gp70 sequence and an N‐terminal part of p15E [27] was cloned into a pET‐22b(+) vector (Merck, Darmstadt, Germany) between the pelB leader and the C‐terminal 6× histidine tag (His tag). The purified plasmid was transformed into *E. coli* BL21‐CodonPlus (DE3)‐RP cells (Agilent, Santa Clara, CA, USA). Recombinant protein production was performed as described above. The bacterial cell pellet was resuspended in PBS, 10 mM DTT, incubated with 1 mg/mL lysozyme, 1000 U benzonase, and complete protease inhibitor EDTA‐free at room temperature for 1 h, and disrupted by sonification as above. The insoluble recombinant protein was pelleted by centrifugation at 50 000 × *g* for 30 min at 4°C. The pellet was resolved in lysis buffer (100 mM NaH_2_PO_4_, 10 mM Tris/HCl; 6 M GuHCl, 10 mM DTT, pH 8.0); incubated at 4°C overnight, centrifuged again at 50 000 × *g* for 30 min at 4°C. The supernatant was filtered through a 0.45 µM filter. Recombinant gp70 was purified by metal chelating affinity chromatography using a Ni‐NTA resin on an ÄKTAprime plus unit (Cytiva Life Sciences, Freiburg, Germany). Both purified proteins were dialyzed against PBS and characterized by SDS‐PAGE and Western blotting.

### 2.3 Goat Antisera

Goat sera against the recombinant p15E (goat serum 355) and gp70 of PERV (goat serum 62) had been prepared using recombinant proteins p15E and gp70 as antigens for the immunization [27].

### 2.4 SDS‐PAGE and Western Blot Analysis

Purified recombinant proteins p15E and gp70 were used as antigens. Sodium dodecyl sulphate‐polyacrylamide gel electrophoresis (SDS‐PAGE) was performed using a 12% gel for gp70 and a 4%–20% gradient gel for p15E. 200 ng of gp70 and 1.3 µg of rp15E were boiled with loading dye for 10 min, followed by SDS‐PAGE run at 80 V. The proteins were transferred to a Roti PVDF membrane 0.2 µm (Carl Roth GmbH, Karlsruhe, Germany) using a semidry transfer apparatus (Peqlab Biotechnologie GmbH, Germany) at 0.13A for 70 min or stained with Imperial Protein Stain (Thermo Fisher Scientific, Waltham, Massachusetts, USA). The blot was incubated in 5% milk powder‐Tris buffered saline with 0.05 % Tween (TBST) blocking buffer for 1 h at room temperature, followed by incubation with positive control sera, goat anti‐gp70 (1:100) or goat anti‐p15E (1:1000) and baboon sera (1:100) overnight at 4°C. The following baboon sera were tested: A (197 days survival), D (14 days survival), E (90 days survival), and F (50 days survival). The blots were incubated with donkey anti‐goat HRP‐coupled IgG (1:20 000) (MilliporeSigma, Massachusetts, USA) or peroxidase AffiniPure goat anti‐human IgG (H+L) (1:10 000) (Jackson ImmunoResearch, West Grove, PA, USA), respectively. The blots were developed using ECL Prime Western blot detection reagent (Cytiva, USA). To demonstrate that the secondary antibody is working, baboon serum was loaded and blotted.

## 3 Results

### 3.1 Method to Screen for PERV RNA

A real‐time RT‐PCR was developed to detect the expression of PERV at the RNA level and to detect the replication of PERV. This real‐time RT‐PCR uses primers located in a highly conserved region of the polymerase (pol) gene of PERV and recognizes both, mRNA and genomic RNA. This PCR recognizes PERV‐A, PERV‐B, and, if present, PERV‐C and PERV‐A/C. The PCR was performed as a duplex PCR, detecting either baboon GAPDH mRNA in the case of baboon tissues or porcine GAPDH mRNA in the case of the explanted pig heart in addition to the PERV pol gene. A PCR control was performed in order to exclude contamination with cellular DNA, which could give a false positive result. As a positive control, RNA obtained from 293 cells producing PERV‐A/C [25] was analyzed.

### 3.2 Absence of PERV Expression in Explanted Pig Hearts and Baboon Organs

Using real‐time RT‐PCR, no PERV‐specific RNA was observed in the baboon organs as well as in the explanted pig heart, indicating absence of PERV mRNA and the absence of viral genomic RNA (Table 1A). Expression of porcine and baboon GAPDH mRNA was found in all samples analyzed. In all baboon organs tested, PERV proviruses had been detected, indicating microchimerism (Table 1A). In the case of baboon A, the expression of PERV had been tested previously; the results correlated with the present results (Table 1A). As a positive control, PERV RNA was found in human 293 cells infected with and producing infectious human‐tropic PERV‐A/C (Table 1B).

**TABLE 1A:** Screening of baboon tissues and an explanted pig heart for PERV proviruses and expression of PERV RNA.

			PCR result	Ct values of RT‐PCR	
Baboon number	Survival time (days)	Tested organ	PERVpol^a^	PERVpol	GAPDH^b^
A	197	Lung	+	n.d./n.d.^c^	20.34/23.54^c^
		Kidney	+	n.d./n.d.^c^	20.44/25.24^c^
		Spleen	+	n.d./n.d.^c^	21.07/21.36^c^
B	195	Liver	+	n.d.	30.58
		Lung	+	n.d.	30.12
		Kidney	+	n.d.	30.70
		Spleen	+	n.d.	29.89
C	169	Liver	+	n.d.	30.10
		Lung	+	n.d.	29.67
		Kidney	+	n.d.	30.24
		Spleen	+	n.d.	30.35
		Heart muscle	+	n.d.	30.02

Abbreviation: n.d., not detected.

^a^ Results of a conventional PCR using primers specific for PERVpol to detect microchimerism, that is proviruses in pig cells.

^b^ Baboon GAPDH was determined with the exception of the explanted pig heart muscle from baboon C, where porcine GAPDH was determined.

^c^ Data measured previously [22].

**TABLE 1B:** Expression of PERV in human PERV‐producing 293 cells.

	Ct values of RT‐PCR	
Cells	PERVpol	GAPDH^a^
293/PERV	26.46	29.08

^a^ Human GAPDH.

**TABLE 2:** Primers and probes used for the real‐time PCRs.

Gen	Primer/probe	Sequence	Location (nucleotide number)	Accession number	Reference
PERV pol	PERV pol fwd	CGACTGCCCCAAGGGTTCAA	3568–3587	GenBank HM159246	Yang et al. [4]
	PERV pol rev	TCTCTCCTGCAAATCTGGGCC	3803–3783		
	PERV pol probe	6FAM‐CACGTACTGGAGGAGGGTCACCTG‐BHQ1	3678–3655		
pGAPDH	pGAPDH fwd	GATCGAGTTGGGGCTGTGACT	1083–1104	GenBank NM_001206359.1	Duvigneau et al. [50]
	pGAPDH rev	ACATGGCCTCCAAGGAGTAAGA	1188–1168		
	pGAPDH probe	HEX‐CCACCAACCCCAGCAAGAGCACGC‐BHQ1	1114–1137		
hGAPDH	hGAPDH fwd	GGCGATGCTGGCGCTGAGTAC	3568–3587	GenBank AF261085	Behrendt et al. [51]
	hGAPDH rev	TGGTTCACACCCATGACGA	3803–3783		
	hGAPDH probe	HEX‐CTTCACCACCATGGAGAAGGCTGGG‐BHQ1	3655–3678		

### 3.3 Method to Screen for PERV‐Specific Antibodies

**FIGURE 1:**
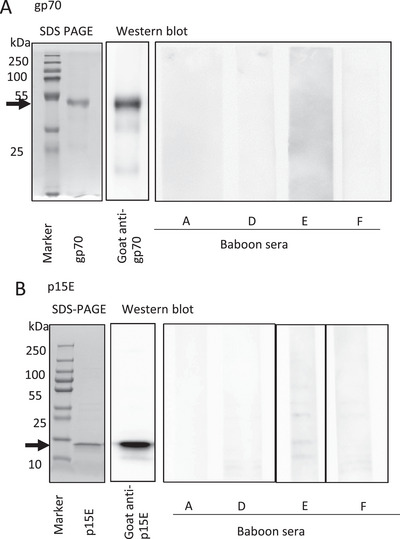
(A) SDS‐PAGE of the purified recombinant gp70 of PERV demonstrating the purity of the antigens used for the Western blot assay and results of the Western blot assays analyzing a goat antiserum as positive control and the baboon sera. (B) the same for recombinant p15E. The arrows indicate the position of recombinant gp70 (p54) and p15E, respectively.

A Western blot analysis was established using freshly produced recombinant p15E and gp70 as antigens and control sera obtained previously by immunization of goats with recombinant p15E and gp70 [27]. The SDS PAGE showed the purity of the recombinant proteins and the Western blot using specific goat sera against the antigens demonstrated the functionality of the assay (Figure 1). By separating the baboon serum by gel electrophoresis, blotting it, and incubating the blot with the secondary antibody, we were able to show that the secondary antibody detected antibodies from the baboon (not shown).

### 3.4 Absence of PERV‐Specific Antibodies in Transplanted Baboon**s**

No PERV‐specific antibodies were detected when a Western blot analysis was performed with sera from baboon A (197 days survival time after transplantation of a pig heart), baboon D (14 days survival), baboon E (90 days survival), and F (50 days survival), using recombinant envelope proteins p15E and gp70 of PERV as antigens. As expected, the assay detected antibodies in the positive control goat sera (Figure 1). This result indicates the absence of a PERV infection. This is the first time that baboon recipients of pig hearts were screened for PERV‐specific antibodies. Previously, Western blot analyses were performed using sera from human xenotransplant recipients [16, 17] or NHPs inoculated with PERV [14, 28].

## 4 Discussion

A real‐time RT‐PCR was established to screen for PERV expression and replication in baboon tissues and in the explanted heart after transplantation of pig hearts. This real‐time RT‐PCR has been used for screening PERV expression in mitogen‐stimulated pig peripheral blood mononuclear cells (PBMCs) and tissues of different pig breeds [29] and in tissues from pigs with melanomas [30]. However, in previous screenings, a primer pair located in the gag sequence was used, whereas in this study, primers targeting a highly conserved sequence in the polymerase gene were used. The fact that we did not detect PERV mRNA and genomic RNA in the explanted pig heart and in baboon tissues indicates that there is no expression and replication of PERV. It is very important to make sure that there is no cellular DNA contamination in the RNA preparation, which would give a false positive result. All tested baboons had been shown to carry pig cells in all organs tested, as previously reported [21, 22]. This microchimerims is typical for xenotransplantation [20]. Microchimerism is the reason why infection of the recipient cannot be easily detected by screening for integrated proviruses. Screening for integrated proviruses by PCR is commonly used to detect infections by exogenous retroviruses, for example, in the case of human immunodeficiency virus (HIV) [31], FeLV [32], or KoRV [33] infections. Since the presence of numerous pig cells makes it difficult to detect the integration of PERV proviruses in the cells of the recipient, other methods have to be used.

No PERV transmission was observed in any clinical or preclinical trials, as well as in inoculation experiments (for a review, see [12]). There have been publications claiming the infection of human and mouse cells [34, 35, 36]. For example, porcine cells that did not produce human‐tropic PERV were transplanted into immunodeficient NOD/SCID (non‐obese diabetic severe combined immunodeficiency) transgenic mice, and human cells from the chimeric mice were frequently found to contain PERV sequences. However, this transmission was attributed to the pseudotyping of PERV‐C (a virus without human tropism) by xenotropic murine leukemia virus, rather than the de novo generation of human‐tropic PERV [37].

It is also important to consider whether PERV can be released from disseminated islet cells that contribute to microchimerism over an extended period. For this reason, long‐term screening for PERV is essential.

Since screening for proviruses is hampered by the presence of disseminated pig cells, which may carry up to 60 proviruses per cell, one effective method to detect infection is to monitor for increasing amounts of PERV mRNA or genomic RNA. This can be achieved using real‐time RT‐PCR or sequencing RNA in the serum or cells of the recipient. In the case of infection of the recipient, PERV expression and replication should be ongoing in the cells of the recipient, not in the pig transplant or in the disseminated pig cells. However, expression and replication of PERV in the transplanted pig organ and the disseminated pig cells can also be harmful to the recipient. Such a process which can be called virus invasion without infection of host cells was observed in the case of the porcine cytomegalovirus, which is a porcine roseolovirus (PCMV/PRV). This virus does not infect human cells, but the unrestricted replication in the transplanted pig organ contributed to the reduction of the survival time of the transplants in NHPs and the first human‐pig heart recipient [18, 21].

Therefore, screening for PERV‐specific antibodies as an indirect indication of virus infection is the method of choice. Most infections by exogenous retroviruses can be diagnosed by the detection of antibodies, for example, HIV infections. When we screened diabetic patients who were treated with encapsulated pig islet cells in New Zealand [16, 38] and Argentina [17] or patients after ex vivo perfusion of bioreactors filled with pig liver cells [39], we used these assays and did not detect antibodies against PERV, indicating that no PERV infection had happened in these clinical trials. In addition to recombinant PERV proteins, purified virus particles have been used as antigens, which allows the detection of antibodies against several viral proteins [17, 39, 40]. Western blot analyses using recombinant PERV proteins and PERV virus particles were also performed when islet cells derived from Göttingen minipigs were transplanted into non‐diabetic cynomolgus monkeys using a macroencapsulation device, and no PERV‐specific antibodies were found [15]. When Paradis et al. [41] analyzed sera from 160 patients who received pig materials for signs of infection using the core protein p27Gag of PERV in Western blot assays, they detected seroreactivity in some samples. When we tested these “positive” sera in our Western blot assays using virus preparations derived from PERV‐producing 293 cells or PK‐15 cells, all samples reacted reproducibly against p27Gag, but none responded against other viral proteins, indicating an absence of PERV infection [40]. The origin of the antibodies against p27Gag is unclear, cross‐reactive antibodies against autoantigens or related retroviruses may be the origin. Cross‐reacting antibodies against the core protein p24 of HIV or HTLV had also been detected in healthy, uninfected humans [42, 43]. Therefore, testing for retrovirus infections using immunological assays should always include at least two different viral proteins.

No antibodies against PERV were found also in preclinical trials [44], some Western blot assays were using lysates of 293 cells infected with PERV [45]. No PERV‐specific antibodies were also detected in virus infection experiments in baboons, rhesus monkeys and pig‐tailed monkeys. The animals were inoculated with high doses of cell‐free virus preparations [14, 28]. For these studies human‐cell adapted PERV‐A/C characterized by an increased virus titer and multimerized repeat sequences in their long terminal repeats (LTR) [2] were inoculated under triple immunosuppression and the Western blot assays indicated absence of infection.

As an alternative to the Western blot analysis, an ELISA can be performed [40]. It is recommended to perform ELISAs based on synthetic peptides. When recombinant proteins were used which are not well purified and still contain proteins from their producing bacteria, antibodies against these bacterial proteins may give false‐positive results. The immunodominant peptide of the transmembrane envelope protein of PERV is an excellent test peptide [40]. Synthetic peptides corresponding to the immunodominant epitope in the transmembrane envelope of HIV‐1 had been used successfully for screening of antibodies against this virus. Nearly 100 % of the infected patients in later clinical stages reacted against this epitope [46, 47]. Synthetic peptides have also been used to screen for antibodies against the human endogenous retrovirus HERV‐K [48]. However, reliable ELISA was also performed using highly purified recombinant proteins [49]. In these studies, no antibodies against PERV were detected in cynomolgus monkeys with diabetes and transplantation of islet cells from Auckland Island pigs [49]. Western blot assays performed in parallel using purified virus particles were also negative.

Since donor pigs for clinical purposes will be carefully screened in advance, xenotransplantation may eventually prove to be safer than allotransplantation, where viruses such as HIV‐1, HCMV, EBV, and the rabies virus have been transmitted. To prevent the transmission of PERVs, which cannot be eliminated as exogenous viruses can, several strategies have been developed. The most practical approaches include a vaccine based on neutralizing antibodies against the envelope proteins [27] or genome editing. Using CRISPR/Cas, all proviruses were inactivated [4], and healthy piglets with inactivated proviruses were successfully generated [5].

## 5 Recommendations

Since microchimerism, that is the presence of pig cells in organs of the recipient complicates the detection of provirus integration, a method typically performed in the case of other retrovirus infections, detection of viral RNA in human cells should be applied for testing for PERV infection.   The detection of PERV‐specific antibodies using Western blot assays or ELISAs has proven to be the method of choice. Recombinant viral proteins, highly purified virus particles, and synthetic peptides corresponding to immunodominant epitopes can be used as antigens in these assays.

## Author Contributions

All authors contributed to the research design, analysis, interpretation of data, and manuscript writing.

## Conflicts of Interest

The authors declare no conflicts of interest.
